# High Seroprevalence for Typhus Group Rickettsiae, Southwestern Tanzania 

**DOI:** 10.3201/eid1902.120601

**Published:** 2013-02

**Authors:** Tatjana Dill, Gerhard Dobler, Elmar Saathoff, Petra Clowes, Inge Kroidl, Elias Ntinginya, Harun Machibya, Leonard Maboko, Thomas Löscher, Michael Hoelscher, Norbert Heinrich

**Affiliations:** Author affiliations: Medical Centre of the University of Munich, Munich, Germany (T. Dill, E. Saathoff, P. Clowes, I. Kroidl, T. Löscher, M. Hoelscher, N. Heinrich);; DZIF German Centre for Infection Research–Ludwig Maximilian University of Munich, Munich (G. Dobler, E. Saathoff, M. Hoelscher, N. Heinrich);; Bundeswehr Institute of Microbiology, Munich (G. Dobler);; NIMR-Mbeya Medical Research Centre, Mbeya, Tanzania (P. Clowes, I. Kroidl, E. Ntinginya, L. Maboko, M. Hoelscher);; Regional Medical Office, Mbeya (H. Machibya)

**Keywords:** typhus group rickettsiae, murine typhus, Tanzania, Mbeya region, vegetation, serology, rickettsia

## Abstract

Rickettsioses caused by typhus group rickettsiae have been reported in various African regions. We conducted a cross-sectional survey of 1,227 participants from 9 different sites in the Mbeya region, Tanzania; overall seroprevalence of typhus group rickettsiae was 9.3%. Risk factors identified in multivariable analysis included low vegetation density and highway proximity.

Murine, or endemic, typhus is primarily caused by *Rickettsia typhi* (typhus group rickettsiae [TGR]) and is usually manifest as a benign disease. A systemic vasculitis causes a clinical triad of fever, headache, and maculopapular rash (*1*). Because these signs and symptoms are nonspecific, the disease is often misdiagnosed or overlooked and can frequently be misclassified as malaria (*2*,*3*). In rare cases, murine typhus can lead to severe systemic complications such as acute renal failure, interstitial pneumonia, and complications of the central nervous system. The case-fatality-rate is <5% (*2*), in contrast to the situation for epidemic, or louse-borne, typhus caused by *R. prowazekii*, which can produce severe disease and fatality rates up to 30% if untreated. Serologic tests cannot distinguish these 2 infections, however. We assume that the antibodies detected in Tanzania in this study were caused by *R. typhi*, because, to our knowledge, no severe or epidemic illness compatible with louse-borne typhus has been described in the study region.

Murine typhus is found throughout the world, widely distributed in subtropical and tropical regions, and is most apparent in port cities with large rat populations (*2*,*4*), which provide a reservoir for the pathogen and its main vector, the rat flea (X*enopsylla cheopsis*). Additional transmission cycles have been described in Texas and California, USA, which involved mainly suburban cats and opossums as reservoir hosts and the cat flea (*Ctenocephalides felis*) as vector (*5*). Other yet unknown cycles may exist.

In Tanzania, information on typhus is sparse. A seroprevalence study among pregnant women from the port city of Dar es Salaam found a seropositivity prevalence of 28% (*4*). In the landlocked northern Tanzanian town of Moshi, murine typhus was detected in 0.5% of febrile patients (*6*).

A predictive risk model for endemic typhus based on environmental conditions has not been established, but because plague is also transmitted by *X. cheopsis* fleas, some of the findings regarding plague transmission might also apply to murine typhus. However, no data are available on the vector flea *C. felis,* the predominant flea harvested from rodents in a study in Uganda (*7*).

## The Study

 We conducted a cross-sectional seroprevalence study among 1,227 persons from the Mbeya region in southwestern Tanzania to estimate TGR seroprevalence rates and to assess associated sociodemographic and environmental risk factors. This study was conducted as a substudy within the EMINI (Evaluation and Monitoring the Impact of New Interventions) longitudinal cohort study. Briefly, in 2005 we conducted a census at 9 study sites (Figure) to collect baseline data, and 10% of census households were chosen by geographically stratified random selection to participate in the 5-year EMINI longitudinal cohort study (http://www.mmrp.org/projects/cohort-studies/emini.html). From these, serum specimens for this substudy were selected by stratified disproportionate random sampling of stored samples from 17,872 persons who took part in the second EMINI survey in 2007. Stratification was done for age (7 categories), altitude of residence (2 categories), and ownership of domestic mammals (2 categories) and resulted in 28 strata of roughly similar size, described in detail elsewhere (*8*). Serum samples were tested for IgG against *R. typhi* by indirect immunofluorescence assay (IIFA) with the same batch of a commercially available test (Rickettsia typhi Spot IF; Fuller Laboratories, Fullerton, CA, USA.). Samples with an IgG titer of >64 or higher were regarded as positive; because IIFA for antibody testing against rickettsiae has a high sensitivity and specificity, as shown by different researchers and with different antigen preparations (*9*). Comparison of the commercial IIFA with a commercial ELISA in our laboratory confirmed this approach (G. Dobler, unpub. data).

**Figure:**
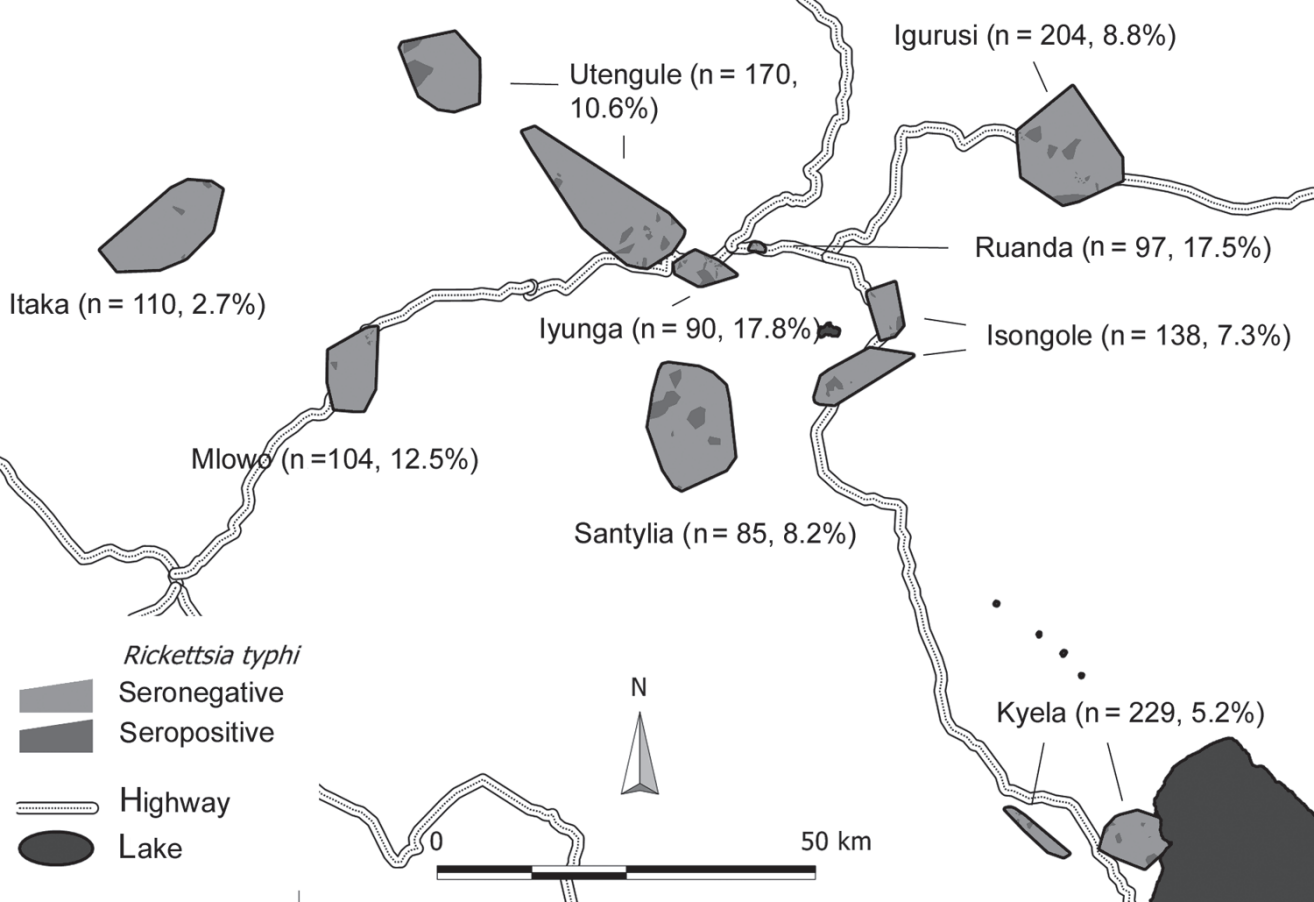
Study sites in Tanzania, showing positivity and negativity for IgG against *Rickettsia typhi* displayed in Voronoi polygons. Every polygon represents 1 household. Numbers in parentheses indicate site prevalence.

To identify possible risk factors for TGR IgG positivity, we analyzed seropositivity as the binary outcome of uni- and multivariable Poisson regression models with robust variance estimates adjusted for household clustering. Initial univariable models for all factors that we deemed as possibly related to TGR infection (Table) were used to identify variables with a univariable p value <0.1 for further multivariable evaluation. Stepwise backward and forward regression, the Akaike and Bayesian information criteria, and various assessments of model fit were used to identify the best multivariable model, in which only variables with a multivariable p value <0.1 were retained.

**Table:** Covariates associated with seropositivity for typhus group rickettsiae, Mbeya region, southwestern Tanzania, 2007*

Covariate/stratum	No. specimens (% positive)	Univariable†‡			Multivariable†§	
		PR (95% CI)	p value		PR (95% CI)	p value
Age, y						
5–<13.6	245 (5.3)	1	–		1 (–)	–
13.6–<27.7	245 (6.5)	1.23 (0.60–2.51)	0.568		1.28 (0.63–2.58)	0.495
27.8–<42.1	243 (12.3)	2.33 (1.23–4.39)	0.009		2.40 (1.28–4.49)	0.006
42.1–55.2	248 (14.9)	2.81 (1.53–5.18)	0.001		2.73 (1.49–4.99)	0.001
>55.2	246 (7.3)	1.38 (0.69–2.76)	0.365		1.41 (0.71–2.80)	0.331
Distance to nearest highway, km	1,227 (9.3)	0.96 (0.94–0.99)	0.012		0.97 (0–0.99)	0.011
Enhanced vegetation index, per 0.1 units	1,227 (9.3)	0.58 (0.44–0.76)	<0.001		0.60 (0.46–0.79)	<0.001
Persons/km^2^, per 1,000 persons	1,227 (9.3)	1.08 (1.04–1.12)	<0.001			
Sex						
F	672 (8.6)	1	–			
M	544 (9.9)	1.15 (0.81–1.63)	0.430			
Unknown	11 (18.2)	2.11 (0.59–7.58)	0.254			
SES rank, per unit¶	1,227 (9.3)	1.08 (1.02–1.15)	0.008			
Cattle/km^2^, per 100 cattle	1,227 (9.3)	1.28 (1.05–1.56)	0.017			
No. cows owned	1,227 (9.3)	0.96 (0.84–1.09)	0.526			
No. goats owned	1,227 (9.3)	0.94 (0.83–1.07)	0.367			
Dogs owned						
No	820 (8.8)	1	**–**			
Yes	191 (8.4)	0.95 (0.54–1.67)	0.869			
No information	216 (12.0)	1.37 (0.89–2.11)	0.150			
Minimum ambient temperature, °C	1,227 (9.3)	0.92 (0.87–0.97)	0.004			
Average day land surface temperature, °C	1,227 (9.3)	1.37 (0.71–2.63)	0.351			
Average night land surface temperature, °C	1,227 (9.3)	0.61 (0.38–0.99)	0.044			
Elevation, per 100 m	1,227 (9.3)	1.04 (1.00–1.08)	0.025			
Rainfall, per 1,000 mm	1,227 (9.3)	0.53 (0.30–0.92)	0.025			

*PR, prevalence ratio; SES, socioeconomic status. Blank cells indicate that the variables were not included in the multivariable analyses due to lack of multivariable significance. †Results of univariable and multivariable Poisson regression adjusted for household clustering by using robust variance estimates. ‡Results of separate models for each of the covariates below. §Multivariable model, including only age, distance to nearest highway, and enhanced vegetation index as covariates. ¶SES rank, rank (from 0 [lowest] to 10 [highest]), according to socioeconomic score.

Of the 1,227 analyzed serum specimens, 114 specimens (9.3%) were positive for TGR IgG. This finding translates into an estimated overall population prevalence of 8.4% (95% CI 6.8%–10.1%) when findings are extrapolated from our stratified sample to the underlying population of the 9 sites by using direct standardization. We found local maximum prevalence in the urban sites, Ruanda (17.5%) and Iyunga (17.8%), and in semiurban Mlowo (12.5%; Figure). The prevalence at other sites ranged from 2.7% to 10.6%. The highest seropositivity rate was found in the age quintile from 42.1 to 55.2 years, with a decline thereafter. In univariable analysis, several environmental covariates showed a significant inverse association with TGR IgG (Table), which included vegetation density, rainfall, minimum and night temperatures, whereas population density, cattle density, and socioeconomic status were positively associated with seropositivity. The geographic distribution of seropositive participants (Figure) led us to include distance to the nearest highway as a variable in the analysis. Distance was found to be inversely associated with seropositivity. The final multivariable model included age, vegetation density, and distance to the nearest highway as significant predictors of TGR IgG. Other factors were not included in the multivariable model because their lack of multivariable significance.

Although significant in univariable analysis, the association of population density, rainfall, socioeconomic status, and cattle density became nonsignificant in the multivariable model when vegetation density was included (p = 0.66 for population density; data not shown). Other factors, including sex, livestock ownership, day and night average land surface temperatures, and other environmental factors, were unrelated to TGR seropositivity.

## Conclusions 

In contrast to results of a recent study of febrile patients from inland northern Tanzania (*6*), site-specific seropositivity prevalences of up to 17.8% in our study suggest that TGR contributes substantially to febrile illness in some areas of the Mbeya region. Our study provides data on environmental risk factors for TGR seropositivity, which might be useful to inform a predictive model for disease occurrence. The inverse association of vegetation density with seropositivity has been described for plague in Uganda, a disease that is also transmitted by the rat flea (*X. cheopsis*) (*10*). That study and early laboratory data suggest that dryness is not the driving factor behind the link between vegetation and disease transmission, because increasingly dry conditions in the laboratory adversely affect vector lifespan (*11*). In our study, rainfall was not significantly associated with seropositivity in the multivariable model. The urbanity of a settlement, expressed by population density and closeness to highways, may still be a relevant factor through providing more favorable habitats for the mammal reservoir hosts, and sparse vegetation could just be a proxy for urbanization. The positive univariable association of seropositivity with socioeconomic status appears to be a product of the higher socioeconomic status in urbanized communities. Our results suggest that TGR incidence may increase with deforestation and increasing urbanization. Additional research is needed to detect the pathogen in acute infection and to describe the local transmission cycle to validate the identified risk factors prospectively. We further hypothesize that remote sensing data could be used to design a model for prediction of *R. typhi* infection, which could be used to direct public health interventions in the future.
